# Treatment preferences in low-risk papillary thyroid microcarcinoma: a discrete choice experiment

**DOI:** 10.3389/fpubh.2025.1686729

**Published:** 2025-12-15

**Authors:** Siming Wang, Jialiang Feng, Jinfeng Yue, Xueqin Niu, Juan He, Yuqi Yang, Jingbo Zhang, Kuo Miao

**Affiliations:** 1 The Fourth Affiliated Hospital of Harbin Medical University, Harbin, China; 2 Zhongnan Hospital of Wuhan University, Wuhan, China; 3 School of Nursing, Henan University of Science and Technology, Luoyang, China; 4 Center for Evidence-Based Chinese Medicine, Beijing University of Chinese Medicine, Beijing, China

**Keywords:** treatment preferences, papillary thyroid microcarcinoma, discrete choice experiment, partial thyroidectomy, thermal ablation, active surveillance

## Abstract

**Objective:**

This study aims to assess treatment preferences among newly diagnosed low-risk papillary thyroid microcarcinoma (PTMC) patients and analyse the trade-offs they make in treatment decisions.

**Design:**

Cross-sectional study.

**Methods:**

Conducted at the Fourth Affiliated Hospital of Harbin Medical University from June 2023 to April 2024, this study employed a discrete choice experiment. Participants were asked to choose among three treatment options with varying levels across seven attributes. A mixed logit model was used to analyse patient preferences, calculate the relative importance (RI) of attributes, and estimate marginal willingness to pay (mWTP). Subgroup analyses were also performed.

**Results:**

The final sample of 418 participants yielded 11,286 observations. The most influential attribute affecting treatment choice was the 10-year risk of disease recurrence or progression (RI = 58.4%), followed by the risk of short-term complications (RI = 8.9%), treatment type (RI = 8.5%), length of hospital stay (RI = 7.7%), need for lifelong thyroid medication (RI = 7.3%), and out-of-pocket treatment costs (RI = 6.9%). The risk of permanent voice change had minimal impact (RI = 2.4%). Regarding treatment type, participants significantly preferred thermal ablation over open surgery, while endoscopic thyroidectomy was less preferred, and active surveillance was the least favoured option. The mWTP analysis reinforced these priorities. Additionally, sociodemographic and psychological factors also influenced preferences.

**Conclusion:**

These findings highlight the need for healthcare providers to clearly communicate the long-term impacts of different treatment options to support informed decision-making. They also underscore the importance of improving patient-centred care, enhancing health education, and addressing the issue of overtreatment in PTMC.

## 1 Introduction

Thyroid cancer is the most common endocrine malignancy, with its global incidence surging over the past three decades, mainly due to advances in imaging and cytological techniques (1, 2). This increase is largely attributed to the detection of papillary thyroid carcinoma (PTC), particularly its subtype, papillary thyroid microcarcinoma (PTMC) (3), defined by the World Health Organization (WHO) (4) as a PTC with a diameter of ≤1 cm. PTMC is often asymptomatic (5), with a very low cancer-specific mortality rate of 0.02% (6), raising concerns about overtreatment linked to PTMC overdiagnosis (7, 8).

Guidelines recommend basing surgical decisions on clinical circumstances and recurrence risk rather than defaulting to total thyroidectomy (TT). Partial thyroidectomy (PT) has now been commonly accepted for the treatment of low-risk PTMC, as it helps preserve thyroid function and reduce the risk of complications (9, 10). And there has been increasing interest in less invasive treatments like endoscopic thyroidectomy (ET) and thermal ablation (TA) compared to traditional open surgery. These methods aim to reduce complications, improve cosmetic outcomes, minimise scarring, shorten hospital stays, and preserve thyroid function while selectively targeting tumours (11, 12). Advances in imaging and surgical tools have expanded the use of ET in China, with various approaches now established, including pre-sternal, axillary, sub clavicular, retro auricular, and transoral methods (13, 14). TA has been shown in large Chinese cohort studies to be an effective and safe treatment for low-risk PTMC after 5 years of follow-up (15, 16).

Active surveillance (AS), a strategy where patients are monitored through regular, detailed check-ups without immediate surgery until disease progression occurs, is commonly used for indolent tumours. AS for PTMC, initiated in Japan in the 1990s (17), and then numerous studies support AS as a suitable initial strategy, with low tumour progression rates and a flexible, cost-effective approach that preserves quality of life (18, 19). A study by Lai et al. (20) found that AS could save 10 × 10^8^ CNY annually for every 50,000 patients in China compared to early surgery. Additionally, a survey of low-risk PTMC patients in Southwest China (June 2016 to June 2021) found that 24.2% (319/1316) of thyroidectomy patients expressed significant regret, mainly due to post-operative declines in quality of life, compared to just 3.4% (4/116) of those undergoing AS. This highlights the importance of comprehensive counselling on treatment risks and outcomes, and the potential value of considering AS as a viable alternative (21).

The complexity of treatment decision-making for PTMC stems from the need to balance effectiveness, invasiveness, and patient preferences. Surgical and ablative treatments, while definitive, pose risks and may affect quality of life, whereas AS can cause psychological burden and uncertainty. Understanding patient preferences is crucial but understudied, with most research from developed countries. China’s unique patient population, healthcare system, and cultural context may lead to different treatment preferences. While studies on PTC and other low-risk TC patients exist, none specifically address low-risk PTMC patients. In 2018, Ahmadi et al. (22) found that low-risk TC patients generally preferred PT over TT, prioritizing risks of cancer recurrence, nerve damage, hypocalcemia, and the need for thyroid hormone supplementation. Similarly, Nickel et al. (23) surveyed 2054 PTC patients, revealing a preference for treatment options with lower follow-up frequency, reduced out-of-pocket costs, fewer voice and calcium issues, and lower risks of invasive thyroid disease and death. A Canadian mixed-methods study highlighted that patients’ choices between AS and early surgery are influenced by perceived risk, long-term health considerations, family and environmental factors, trust in healthcare providers, and personal preferences regarding surgery (24). Research on patient preferences for ET and TA remains scarce.

Currently, there is limited research on the treatment preferences of low-risk PTMC patients in China, particularly regarding comparisons between PT, TA, and AS based on the latest clinical guidelines and treatment attributes. To address this gap, this study aims to examine factors influencing patients’ treatment decisions and how they weigh different trade-offs. Understanding these factors will contribute to patient-centred care, optimise healthcare resource allocation, and ultimately improve treatment outcomes and patient satisfaction.

## 2 Materials and methods

Given the effectiveness of Discrete Choice Experiments (DCEs) in predicting real-world behaviour, particularly in healthcare (25, 26), this study used a DCE to explore treatment preferences among participants at the Department of Ultrasound, Fourth Affiliated Hospital of Harbin Medical University, from June 2023 to April 2024. DCEs assess participants’ preferences by presenting alternative options defined by various attributes and levels. This study was approved by the hospital’s ethics board (2022-WZYSLLSC-37), with all participants providing written informed consent before completing anonymous electronic or paper-based surveys. No identifiable patient information was collected. The study adhered to the International Society for Pharmacoeconomics and Outcomes Research (ISPOR) (27) guidelines for DCE in healthcare and the American Association for Public Opinion Research (AAPOR) (28) reporting standards.

### 2.1 Development of attributes and levels

We first conducted a literature review to identify all factors influencing treatment decisions for low-risk PTMC patients. Due to the potential complexity this could introduce to the DCE, we conducted one-on-one interviews with 10 low-risk PTMC patients and used a two-round Delphi method with two methodology experts and four clinical experts to refine the attributes. Ultimately, seven key attributes were selected, and attribute levels were tailored to local conditions in China, with evenly distributed levels to better estimate linear relationships (Supplementary Table S1). Different levels were set for each treatment option to enhance the realism of the choice task.

### 2.2 Experimental design and questionnaire development

We used Ngene to create an orthogonal design, generating 36 choice tasks. To reduce cognitive load, we divided these tasks into 4 blocks of 9 tasks each and randomly assigned participants to 1 block. Participants completed a pre-experimental practice and test task, resulting in a total of 10 choice tasks per participant. They chose between three treatment options: PT, TA, and AS. To prevent terminology-induced fear and preconceived biases, the DCE scenarios used the term ‘nodule’ and presented options as Treatment Plan A (PT) and B (TA), thereby ensuring that patient preferences were based on attribute trade-offs (Supplementary Table S2). A pilot study was conducted in May 2023 with 80 participants, leading to revisions for clarity and acceptability. The final questionnaire comprised the DCE section and collected comprehensive information on participants’ sociodemographic characteristics (age, gender, living area, income, and education level), clinical features (nodule risk classification), and assessed participants’ psychological characteristics, including scores of General Self-Efficacy Scale (GSES) (29), Perceived Social Support Scale (PSSS) (30), and Fear of Progression Questionnaire-Short Form (FoP-Q-SF) (31) as well as Health Literacy Scale-Short Form (HLS-SF) (32) (Supplementary Table S3).

The GSES assesses individuals’ perceived capability to overcome challenges. This 3-item tool uses a 5-point Likert scale (1 = Strongly disagree to 5 = Strongly agree), yielding total scores from 5 to 15. Higher scores indicate greater problem-solving confidence. In this study, the GSES demonstrated excellent reliability (Cronbach’s *α* = 0.9270).

The PSSS measures perceived support adequacy. Participants rate 3 items using a 7-point Likert scale (1 = Strongly disagree to 7 = Strongly agree), generating total scores from 3 to 21. Elevated scores denote stronger perceived social support. The PSSS exhibited strong reliability in this research (Cronbach’s *α* = 0.7824).

The FoP-Q-SF assesses progression-related anxieties. Participants rate all 12 items using a 5-point Likert scale (1 = Never to 5 = Very often), yielding total scores ranging from 12 to 60. The scale demonstrated excellent reliability in this study (Total Cronbach’s *α* = 0.9121).

The HLS-SF assesses individuals’ health literacy levels. This 4-item tool uses a 4-point Likert scale (1 = Very difficult to 4 = Very easy), yielding total scores from 4 to 16. Higher scores indicate better health literacy. In this study, the HLS-SF demonstrated excellent reliability (Cronbach’s *α* = 0.9257).

### 2.3 Target population and sample size

The target population for this study comprised newly diagnosed low-risk PTMC patients who have not yet received treatment, and they could receive any of the PT, TA, and AS treatment regimens according to the guidelines. The following criteria were used to recruit patients for this study. Inclusion Criteria: (1) Age≥18 years; (2) differentiated thyroid cancer with ultrasound malignancy risk stratification of 4A, 4B, or 4C (10), and a solitary nodule; (3) no treatment or active surveillance at the time of initial thyroid nodule diagnosis; (4) ability to independently complete the questionnaires; and (5) voluntary participation in the survey with signed informed consent. Exclusion criteria: (1) Patients for whom TA or AS is not recommended, including those with a family history of thyroid cancer (parents, siblings, or children), history of radiation exposure to the head and neck, cervical lymph node metastasis, distant metastasis, or extrathyroidal invasion (e.g., trachea, oesophagus, carotid artery, or mediastinal invasion), primary lesions with a maximum diameter >1 cm or bilateral lesions, or the presence of clinical/imaging features highly suggestive of aggressive pathological subtypes, such as tall cell papillary carcinoma, columnar cell type, diffuse sclerosing type, solid subtype, extensively invasive follicular carcinoma, or poorly differentiated thyroid carcinoma. (2) Patients for whom surgery is not recommended, including those with suspected tracheal stenosis, difficult retrosternal goitre, or local invasion (e.g., laryngeal trachea, oesophagus, nerves, blood vessels, upper mediastinum, or extensive skin involvement), or poor tolerance to anaesthesia and surgery, such as those with poorly controlled ASA grade II and above comorbidities, patients on dialysis, patients on anticoagulation or antiplatelet therapy, patients with epilepsy, anxiety, obstructive sleep apnoea, hearing or visual impairment, psychiatric disorders, or pregnant women.

The method proposed by Johnson (33) and Orme (34) is utilised to calculate the sample size (*N*), which depends on the number of choice tasks (*t*), the number of alternatives ( *a* ), and the maximum number of levels across different attributes (*c*) in the DCE. The calculation formula is *N* > 500 × *c*/(*t* × *a*). In our study, the values for *c*, *t*, and *a* are 4, 9 (excluding the test choice set), and 3, respectively. Therefore, the estimated *N* > 500 × 4/(9 × 3) ≈ 75. We administered four versions of questionnaires, resulting in a total sample size of no less than 300.

### 2.4 Data collection and quality

Trained surveyors conducted face-to-face data collection in the hospital’s ultrasound department. Patients were informed that this was a DCE, not a physician-guided tool for treatment decisions. Participants completed paper or electronic questionnaires, with some requiring surveyors to read or explain questions. Duplicate responses, incomplete paper questionnaires, and those completed in under 5 min were excluded to ensure data reliability.

### 2.5 Statistical analysis

Data analysis was conducted using Microsoft Excel, RStudio 2024.04.2 and Stata 18. Characteristics of participants were summarised using frequencies and percentages for categorical variables, and as means with standard deviations (SD) for approximately normally distributed continuous variables or medians with interquartile ranges (IQR) for skewed distributions. A mixed logit model (MLM) with 500 Halton draws was employed to analyse treatment preferences among patients with low-risk PTMC, treating all attributes as random parameters. Model goodness of fit was evaluated using the McFadden pseudo *R*^2^. Results from the mixed logit model were then used to calculate the relative importance (RI) of attributes and marginal willingness to pay (mWTP), which was computed for a change in each attribute level using the formula: mWTP = − (β_level/β_cost) where $\beta_{\text{level}}$ is the coefficient of the attribute level, and $\beta_{\text{cost}}$ is the coefficient of the cost attribute (35). Subgroup analyses were conducted by age, gender, residence, income, education, nodule risk, and scores on health literacy (HL), self-efficacy (SE), social support (SS), and disease fear of progression (FoP); *p* < 0.05 was considered unlikely that the observed differences or effects were due to random chance.

## 3 Results

### 3.1 Respondent characteristics

**Figure 1:**
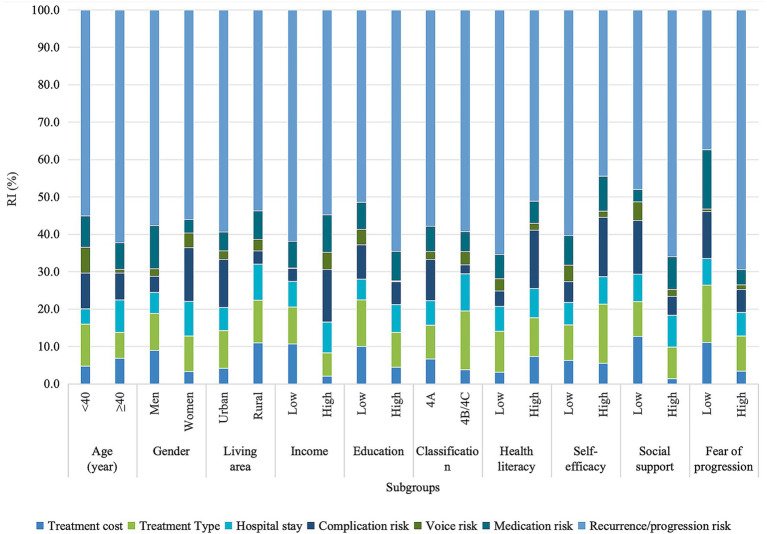
Study population selection flowchart.

Out of 500 eligible participants, 82 (16.4%) were excluded from the survey. The sample selection process is illustrated in Figure 1. The final analysis included 418 participants (83.6%), providing 11,286 DCE observations. Table 1 outlines the characteristics of these participants. The mean (SD) age of them was 43.9 (14.0) years, with a median of 40 years (IQR: 33–53). Women accounted for 53.4% of the sample and 80.6% lived in urban areas. Over half (53.6%) reported a monthly per capita household income below 10,000 CNY, and 67.2% had college-level education or above. Most participants (76.6%) were classified as having 4A risk nodules. The median HLS-SF score was 12 (IQR: 11–12), GSES was 12 (IQR: 12–13), PSSS was 17 (IQR: 16–18), and FoP-Q-SF was 29 (IQR: 19–36).

**Table 1:** Characteristics of participants included in the analysis (*n* = 418).

Variables	*N* (%)/median (IQR)
Age groups (year)	40 (33–53)
<40	169 (40.4)
≥40	249 (59.6)
Gender	
Men	195 (46.7)
Women	223 (53.4)
Living area	
Urban (city)	337 (80.6)
Rural (town/countryside)	81 (19.4)
Monthly per capita household income (CNY)	
<10,000	224 (53.6)
≥10,000	194 (46.4)
Education levels	
Low (junior/senior high school or below)	137 (32.8)
High (college/bachelor’s degree or above)	281 (67.2)
Nodule risk classification	
4A	320 (76.6)
4B/4C	98 (23.4)
HLS-SF scores (point)	12 (11–12)
Low (<12)	132 (31.6)
High (≥12)	286 (68.4)
GSES scores (point)	12 (12–13)
Low (≤12)	280 (67.0)
High (>12)	138 (33.0)
PSSS scores (point)	17 (16–18)
Low (≤17)	211 (50.5)
High (>17)	207 (49.5)
FoP-Q-SF scores (point)	29 (19–36)
Low (<29)	205 (49.0)
High (≥29)	213 (51.0)

IQR, interquartile range; HLS-SF, Health Literacy Scale-Short Form; GSES, General Self-Efficacy Scale; PSSS, Perceived Social Support Scale; FoP-Q-SF, Fear of Progression Questionnaire-Short Form.

### 3.2 Main effects

Table 2 shows the MLM results of the main survey, showing that 6 treatment attributes significantly influenced preferences. The most influential attribute was the risk of disease recurrence/progression within 10 years (*β* = −0.42, 95% CI: −0.48 to −0.37; RI = 58.4%). Other attributes with notable impact included the risk of short-term complications (*β* = −0.04, 95% CI: −0.06 to −0.02; RI = 8.9%), treatment type (RI = 8.5%), length of hospital stay (*β* = −0.18, 95% CI: −0.25 to −0.10; RI = 7.7%), and risk of requiring lifelong thyroid replacement medication (*β* = −0.02, 95% CI: −0.04 to −0.01; RI = 7.3%). Out-of-pocket treatment cost also significantly affected preferences (*β* = −0.05, 95% CI: −0.07 to −0.03; RI = 6.9%). For treatment type, TA was significantly preferred over open surgery (*β* = 1.10, 95% CI: 0.50 to 1.70), whereas ET was moderately preferred (*β* = −0.43, 95% CI: −0.80 to −0.06). In contrast, AS was significantly less preferred compared to open surgery (*β* = −1.17, 95% CI: −2.01 to −0.33). The risk of permanent voice change did not significantly influence preferences (RI = 2.4%).

**Table 2:** Main effects and mWTP from the mixed nested logit model (*n* = 418).

Attributes/levels	Attributes/levels	*β* (95% CI)	*p*	RI (%)	mWTP (95% CI)
Out-of-pocket costs for treatment (1,000 CNY)	Treatment cost (1,000 CNY)	−0.05 (−0.07 to −0.03)	<0.001	6.9	NA
Treatment type (anaesthesia + scar)	Treatment type			8.5	
Open surgery (general anaesthesia + neck scar)	Open surgery	Reference			Reference
Endoscopic surgery (general anaesthesia + axillary/chest scar)	Endoscopic surgery	−0.43 (−0.80 to −0.06)	0.02		−9.10 (−17.81 to −0.40)
Thermal ablation (local anaesthesia + no visible scar)	Thermal ablation	1.10 (0.50 to 1.70)	<0.001		23.31 (6.71 to 39.92)
Active surveillance (no anaesthesia + no visible scar)	Active surveillance	−1.17 (−2.01 to −0.33)	0.01		−24.73 (−42.09 to −7.38)
Length of hospital stays (1 day)	Hospital stay (1 day)	−0.18 (−0.25 to −0.10)	<0.001	7.7	−3.72 (−5.96 to −1.48)
Risk of short-term complications (1%)	Complication risk (1%)	−0.04 (−0.06 to −0.02)	0.001	8.9	−0.86 (−1.45 to −0.27)
Risk of permanent voice change (1%)	Voice risk (1%)	0.07 (−0.03 to 0.16)	0.2	2.4	1.38 (−0.85 to 3.61)
Risk of requiring lifelong thyroid replacement medication (1%)	Medication risk (1%)	−0.02 (−0.04 to −0.01)	0.01	7.3	−0.53 (−0.93 to −0.12)
Risk of disease recurrence/progression within 10 years (1%)	Recurrence/progression risk (1%)	−0.42 (−0.48 to −0.37)	<0.001	58.4	−8.94 (−12.53 to −5.36)

mWTP, marginal willingness to pay; CI, confidence interval; SE, standard error; RI, relative importance; NA, not applicable. Model fit: log likelihood = −2433.1, McFadden *R*^2^ = 0.33 (11,286 observations).

### 3.3 Marginal willingness to pay

The results of the mWTP analysis are presented in Table 2. Participants demonstrated a strong preference for reducing the risk of disease recurrence/progression, with an mWTP of 8.94 CNY (95% CI: 5.36 to 12.53) for a 1% reduction. They were also willing to pay 3.72 CNY (95% CI: 1.48 to 5.96) to shorten hospital stay by 1 day, and 0.86 CNY (95% CI: 0.27 to 1.45) to reduce the risk of short-term complications by 1%. For treatment type, participants were willing to pay 23.31 CNY (95% CI: 6.71 to 39.92) to receive TA over open surgery. In contrast, they were less willing to choose AS, with a negative mWTP of −24.73 CNY (95% CI: −42.09 to −7.38), and showed a modest dispreference for ET (mWTP = −9.10 CNY, 95% CI: −17.81 to −0.40). The mWTP to avoid lifelong thyroid medication was modest but significant (0.53 CNY, 95% CI: 0.12 to 0.93), whereas no significant mWTP was observed for the risk of permanent voice change (1.38 CNY, 95% CI: −0.85 to 3.61).

### 3.4 Subgroup analysis

**Figure 2:**
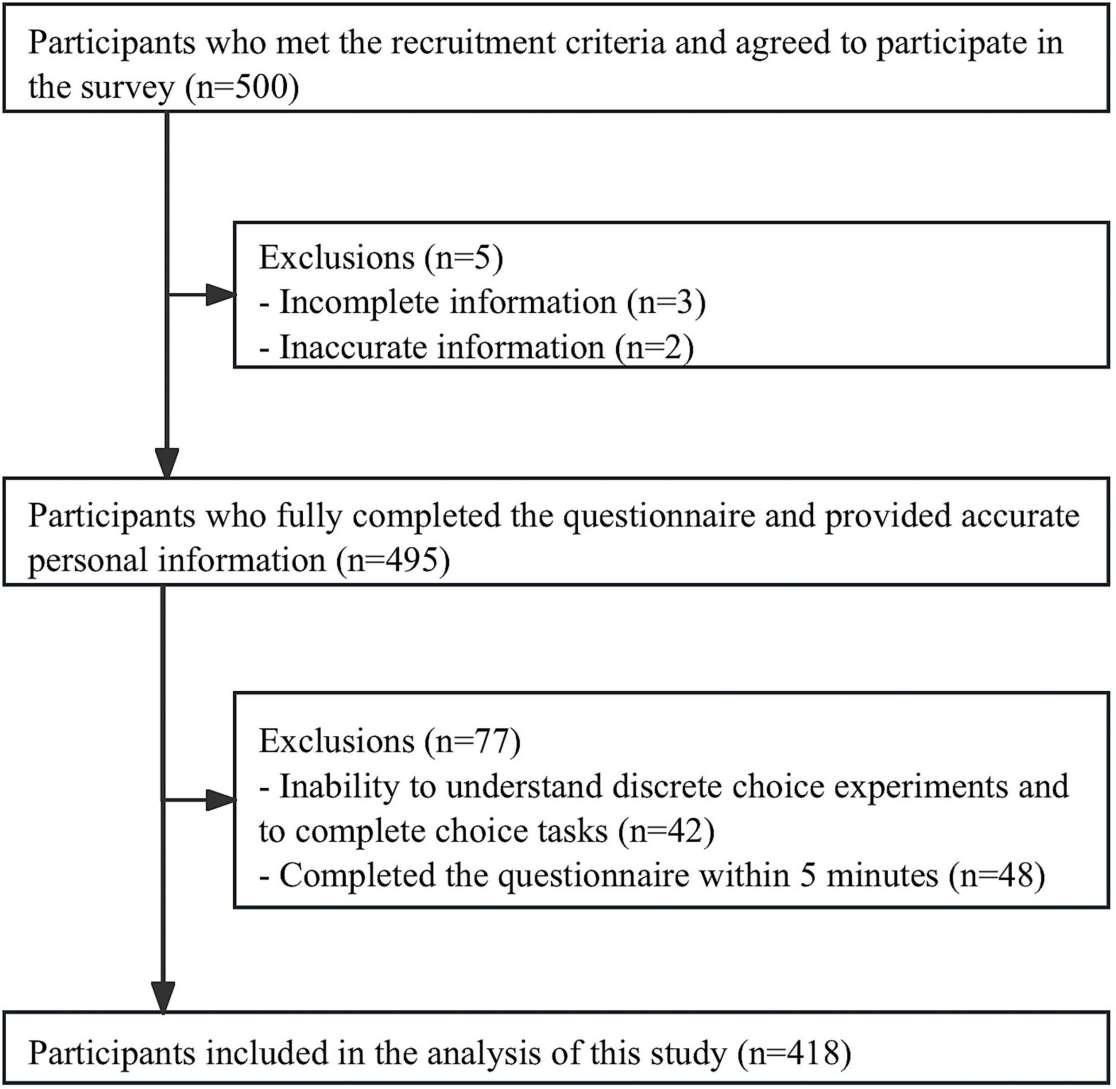
RI for the attributes of subgroups. RI, relative importance.

**Figure 3:**
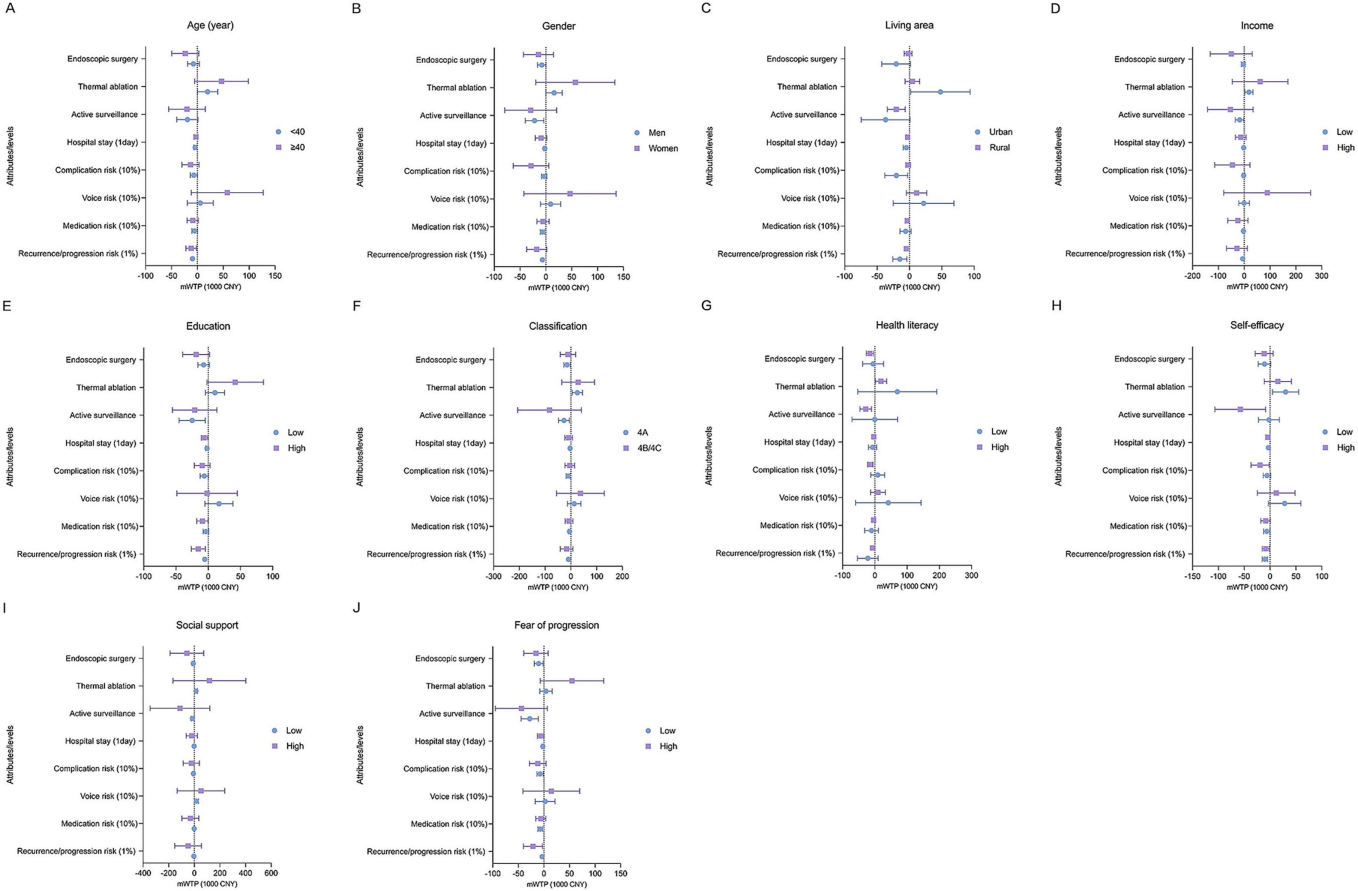
mWTP for subgroups. mWTP, marginal willingness to pay. **(A)** mWTP of the subgroup population older than 40 years and younger than 40 years. **(B)** mWTP of the gender subgroup. **(C)** mWTP of people from different residential areas. **(D)** mWTP of people with different incomes. **(E)** mWTP of people with different levels of education. **(F)** mWTP of people at different stages. **(G)** mWTP of people with different health literacy levels. **(H)** mWTP of people with different self-efficacy levels. **(I)** mWTP of people with different levels of social support. **(J)** mWTP of people with different levels of fear of progression.

Subgroup analysis (Table 3; Figures 2, 3) showed significant variation in treatment preferences, attribute RI and mWTP across demographic and socio-economic groups.

**Table 3:** Results of subgroup analysis.

Attributes/levels	Age (year)				Gender			
	<40 (*n* = 169)		≥40 (*n* = 249)		Men (*n* = 195)		Women (*n* = 223)	
	*β* (95% CI)	*p*	*β* (95% CI)	*p*	*β* (95% CI)	*p*	*β* (95% CI)	*p*
Treatment cost (1,000 CNY)	−0.05 (−0.08 to −0.02)	<0.001	−0.03 (−0.06 to 0.00)	0.02	−0.07 (−0.10 to −0.04)	<0.001	−0.02 (−0.05 to 0.00)	0.08
Treatment type								
Open surgery	Reference		Reference		Reference		Reference	
Endoscopic surgery	−0.38 (−0.92 to 0.17)	0.2	−0.77 (−1.36 to −0.17)	0.01	−0.53 (−1.05 to −0.01)	0.05	−0.33 (−0.88 to 0.23)	0.3
Thermal ablation	0.99 (0.19 to 1.78)	0.02	1.52 (0.55 to 2.49)	0.002	1.06 (0.18 to 1.94)	0.02	1.29 (0.45 to 2.12)	0.002
Active surveillance	−0.96 (−2.00 to 0.07)	0.07	−0.66 (−1.84 to 0.53)	0.3	−1.47 (−2.73 to −0.21)	0.02	−0.66 (−1.75 to 0.42)	0.2
Hospital stay (1 day)	−0.21 (−0.31 to −0.12)	<0.001	−0.09 (−0.21 to 0.02)	0.1	−0.14 (−0.25 to −0.03)	0.01	−0.21 (−0.32 to −0.10)	<0.001
Complication risk (1%)	−0.03 (−0.06 to 0.00)	0.02	−0.04 (−0.08 to −0.01)	0.02	−0.02 (−0.05 to 0.01)	0.2	−0.07 (−0.10 to −0.03)	<0.001
Voice risk (1%)	0.03 (−0.09 to 0.15)	0.7	0.19 (0.05 to 0.32)	0.006	0.06 (−0.06 to 0.18)	0.3	0.11 (−0.03 to 0.25)	0.1
Medication risk (1%)	−0.03 (−0.05 to 0.00)	0.05	−0.03 (−0.06 to 0.00)	0.06	−0.04 (−0.07 to −0.01)	0.003	−0.01 (−0.04 to 0.01)	0.3
Recurrence/progression risk (1%)	−0.47 (−0.56 to −0.39)	<0.001	−0.40 (−0.48 to −0.31)	<0.001	−0.45 (−0.54 to −0.37)	<0.001	−0.40 (−0.47 to −0.33)	<0.001
Observations	4,563		6,723		5,265		6,021	
Log likelihood	−964.7		−1455.2		−1118.9		−1294.0	
McFadden *R*^2^	0.31		0.34		0.30		0.36	

Attributes/levels	Living area				Income			
	Urban (*n* = 337)		Rural (*n* = 81)		Low (*n* = 224)		High (*n* = 194)	
	*β* (95% CI)	*p*	*β* (95% CI)	*p*	*β* (95% CI)	*p*	*β* (95% CI)	*p*
Treatment cost (1,000 CNY)	−0.03 (−0.05 to −0.01)	0.008	−0.15 (−0.22 to −0.08)	<0.001	−0.07 (−0.09 to −0.04)	<0.001	−0.02 (−0.04 to 0.01)	0.2
Treatment type								
Open surgery	Reference		Reference		Reference		Reference	
Endoscopic surgery	−0.54 (−0.96 to −0.11)	0.01	−0.32 (−1.21 to 0.57)	0.5	−0.24 (−0.74 to 0.26)	0.3	−0.82 (−1.43 to −0.22)	0.008
Thermal ablation	1.24 (0.56 to 1.92)	<0.001	0.65 (−0.92 to 2.21)	0.4	1.21 (0.35 to 2.07)	0.006	1.01 (0.17 to 1.84)	0.02
Active surveillance	−0.96 (−1.88 to −0.04)	0.04	−3.02 (−5.07 to −0.97)	0.004	−1.18 (−2.28 to −0.09)	0.03	−0.88 (−2.03 to 0.27)	0.1
Hospital stay (1 day)	−0.13 (−0.21 to −0.05)	0.001	−0.43 (−0.67 to −0.2)	<0.001	−0.14 (−0.25 to −0.03)	0.01	−0.22 (−0.33 to −0.11)	<0.001
Complication risk (1%)	−0.05 (−0.08 to −0.03)	<0.001	−0.03 (−0.09 to 0.02)	0.3	−0.01 (−0.05 to 0.02)	0.4	−0.08 (−0.11 to −0.04)	<0.001
Voice risk (1%)	0.06 (−0.05 to 0.16)	0.3	0.16 (−0.04 to 0.37)	0.1	0.00 (−0.14 to 0.13)	1.0	0.15 (0.00 to 0.29)	0.04
Medication risk (1%)	−0.02 (−0.04 to 0)	0.1	−0.05 (−0.10 to 0.00)	0.04	−0.02 (−0.05 to 0.00)	0.07	−0.04 (−0.07 to −0.01)	0.009
Recurrence/progression risk (1%)	−0.39 (−0.45 to −0.33)	<0.001	−0.75 (−0.99 to −0.52)	<0.001	−0.4 (−0.48 to −0.32)	<0.001	−0.46 (−0.55 to −0.38)	<0.001
Observations	9,099		2,187		6,048		5,238	
Log likelihood	−1959.6		−430.8		−1316.5		−1093.5	
McFadden *R*^2^	0.33		0.38		0.34		0.33	

Attributes/levels	Education				Classification			
	Low (*n* = 137)		High (*n* = 281)		4A (*n* = 320)		4B/4C (*n* = 98)	
	*β* (95% CI)	*p*	*β* (95% CI)	*p*	*β* (95% CI)	*p*	*β* (95% CI)	*p*
Treatment cost (1,000 CNY)	−0.08 (−0.13 to −0.04)	<0.001	−0.03 (−0.05 to −0.01)	0.007	−0.05 (−0.07 to −0.03)	<0.001	−0.02 (−0.06 to 0.01)	0.2
Treatment type								
Open surgery	Reference		Reference		Reference		Reference	
Endoscopic surgery	−0.59 (−1.30 to 0.12)	0.1	−0.53 (−1.00 to −0.07)	0.03	−0.70 (−1.21 to −0.20)	0.007	−0.27 (−0.9 to 0.36)	0.4
Thermal ablation	0.87 (−0.23 to 1.98)	0.1	1.19 (0.42 to 1.97)	0.003	1.19 (0.48 to 1.91)	0.001	0.69 (−0.35 to 1.72)	0.2
Active surveillance	−2.07 (−3.62 to −0.52)	0.009	−0.60 (−1.62 to 0.43)	0.3	−1.27 (−2.26 to −0.28)	0.01	−2.01 (−3.67 to −0.35)	0.02
Hospital stay (1 day)	−0.15 (−0.30 to −0.01)	0.04	−0.16 (−0.25 to −0.07)	<0.001	−0.16 (−0.24 to −0.07)	<0.001	−0.21 (−0.38 to −0.04)	0.01
Complication risk (1%)	−0.05 (−0.10 to −0.01)	0.03	−0.03 (−0.05 to 0.00)	0.07	−0.05 (−0.08 to −0.02)	<0.001	−0.01 (−0.05 to 0.03)	0.6
Voice risk (1%)	0.14 (−0.02 to 0.30)	0.09	0.00 (−0.14 to 0.13)	0.9	0.06 (−0.05 to 0.18)	0.3	0.09 (−0.07 to 0.25)	0.2
Medication risk (1%)	−0.03 (−0.07 to 0.01)	0.1	−0.03 (−0.05 to 0.00)	0.02	−0.02 (−0.05 to 0.00)	0.03	−0.02 (−0.05 to 0.01)	0.2
Recurrence/progression risk (1%)	−0.45 (−0.56 to −0.34)	<0.001	−0.44 (−0.51 to −0.37)	<0.001	−0.43 (−0.50 to −0.36)	<0.001	−0.4 (−0.51 to −0.29)	<0.001
Observations	3,699		7,587		8,640		2,646	
Log likelihood	−798.67		−1613.3		−1808.4		−611.92	
McFadden *R*^2^	0.36		0.32		0.34		0.28	

Attributes/levels	Health literacy				Self-efficacy			
	Low (*n* = 132)		High (*n* = 286)		Low (*n* = 280)		High (*n* = 138)	
	*β* (95% CI)	*p*	*β* (95% CI)	*p*	*β* (95% CI)	*p*	*β* (95% CI)	*p*
Treatment cost (1,000 CNY)	−0.02 (−0.05 to 0.01)	0.2	−0.05 (−0.08 to −0.03)	<0.001	−0.05 (−0.07 to −0.02)	<0.001	−0.04 (−0.07 to −0.01)	0.01
Treatment type								
Open surgery	Reference		Reference		Reference		Reference	
Endoscopic surgery	−0.12 (−0.80 to 0.57)	0.7	−0.83 (−1.32 to −0.35)	0.001	−0.49 (−1.02 to 0.04)	0.07	−0.47 (−1.09 to 0.16)	0.1
Thermal ablation	1.48 (0.32 to 2.63)	0.01	1.02 (0.27 to 1.77)	0.007	1.38 (0.54 to 2.21)	0.001	0.62 (−0.29 to 1.53)	0.2
Active surveillance	0.00 (−1.51 to 1.50)	1.0	−1.52 (−2.48 to −0.56)	0.002	−0.10 (−1.04 to 0.84)	0.8	−2.36 (−3.81 to −0.90)	0.002
Hospital stay (1 day)	−0.15 (−0.29 to −0.01)	0.03	−0.19 (−0.28 to −0.10)	<0.001	−0.15 (−0.25 to −0.05)	0.004	−0.18 (−0.30 to −0.06)	0.003
Complication risk (1%)	0.02 (−0.02 to 0.06)	0.3	−0.08 (−0.11 to −0.04)	<0.001	−0.03 (−0.06 to 0.00)	0.08	−0.08 (−0.12 to −0.04)	<0.001
Voice risk (1%)	0.09 (−0.06 to 0.24)	0.3	0.05 (−0.07 to 0.17)	0.4	0.13 (0.01 to 0.25)	0.03	0.05 (−0.09 to 0.19)	0.5
Medication risk (1%)	−0.02 (−0.06 to 0.01)	0.2	−0.02 (−0.05 to 0.00)	0.07	−0.03 (−0.06 to 0.00)	0.03	−0.04 (−0.06 to −0.01)	0.02
Recurrence/progression risk (1%)	−0.47 (−0.58 to −0.36)	<0.001	−0.39 (−0.46 to −0.33)	<0.001	−0.46 (−0.54 to −0.38)	<0.001	−0.35 (−0.43 to −0.27)	<0.001
Observations	3,564		7,722		7,560		3,726	
Log likelihood	−715.79		−1674.9		−1598.2		−813.67	
McFadden *R*^2^	0.37		0.32		0.35		0.30	

Attributes/levels	Social support				Fear of progression			
	Low (*n* = 211)		High (*n* = 207)		Low (*n* = 205)		High (*n* = 213)	
	*β* (95% CI)	*p*	*β* (95% CI)	*p*	*β* (95% CI)	*p*	*β* (95% CI)	*p*
Treatment cost (1,000 CNY)	−0.09 (−0.12 to −0.05)	<0.001	−0.01 (−0.03 to 0.01)	0.4	−0.08 (−0.11 to −0.04)	<0.001	−0.03 (−0.05 to 0.00)	0.03
Treatment type								
Open surgery	Reference		Reference		Reference		Reference	
Endoscopic surgery	−0.74 (−1.46 to −0.03)	0.04	−0.58 (−1.07 to −0.09)	0.02	−0.79 (−1.38 to −0.20)	0.008	−0.41 (−0.94 to 0.11)	0.1
Thermal ablation	1.11 (0.27 to 1.95)	0.010	1.18 (0.31 to 2.04)	0.008	0.31 (−0.58 to 1.20)	0.5	1.46 (0.67 to 2.25)	<0.001
Active surveillance	−1.26 (−2.37 to −0.15)	0.03	−1.12 (−2.38 to 0.15)	0.08	−2.10 (−3.32 to −0.89)	0.001	−1.17 (−2.47 to 0.13)	0.08
Hospital stay (1 day)	−0.17 (−0.28 to −0.06)	0.002	−0.20 (−0.31 to −0.09)	<0.001	−0.16 (−0.27 to −0.05)	0.003	−0.16 (−0.26 to −0.07)	0.001
Complication risk (1%)	−0.07 (−0.10 to −0.03)	<0.001	−0.02 (−0.06 to 0.01)	0.2	−0.06 (−0.09 to −0.02)	0.003	−0.03 (−0.06 to 0.00)	0.05
Voice risk (1%)	0.13 (0.00 to 0.27)	0.06	0.05 (−0.07 to 0.17)	0.4	0.02 (−0.13 to 0.16)	0.8	0.04 (−0.10 to 0.17)	0.6
Medication risk (1%)	−0.01 (−0.03 to 0.01)	0.3	−0.03 (−0.06 to 0.00)	0.02	−0.05 (−0.08 to −0.02)	<0.001	−0.02 (−0.04 to 0.01)	0.2
Recurrence/progression risk (1%)	−0.34 (−0.42 to −0.27)	<0.001	−0.49 (−0.57 to −0.40)	<0.001	−0.27 (−0.33 to −0.21)	<0.001	−0.57 (−0.67 to −0.47)	<0.001
Observations	5,697		5,589		5,535		5,751	
Log likelihood	−1321.9		−1066.5		−1265.8		−1126.4	
McFadden *R*^2^	0.32		0.35		0.31		0.35	

## 4 Discussion

### 4.1 Treatment attributes influencing preferences

This study provides critical insights into the treatment preferences of Chinese patients newly diagnosed with low-risk PTMC. Among the evaluated attributes, the 10-year risk of disease recurrence/progression emerged as the most influential factor guiding patient decision-making, and the mWTP analysis reinforced this prioritization. This finding underscores a prevailing patient focus on long-term outcomes and a strong desire for clinical reassurance, even when such assurance entails accepting more invasive treatments or higher financial costs. Beyond this, patients also placed considerable weight on short-term complications, hospital stay, and out-of-pocket costs. This reflects a nuanced decision-making process, where individuals balance long-term clinical benefits against short-term recovery burdens and economic implications. It is noteworthy that overdiagnosis and overtreatment of PTMC in China have become a significant public health issue, affecting millions of patients annually and placing a heavy burden on the healthcare system (36). Nevertheless, our study reveals a continued public mistrust of non-invasive approaches. TA emerged as the most preferred treatment, indicating growing acceptance of minimally invasive, intervention-based, and quicker recovery options. In contrast, AS was the least favoured, underscoring persistent discomfort with conservative management strategies, even though these are clinically appropriate for many low-risk cases.

### 4.2 Patient characteristics influencing preferences

Our study found that patients who preferred TA were typically well-educated urban residents with a strong FoP. These individuals, often with higher cognitive capacity and greater concern for quality of life (37), tended to favour a minimally invasive, rapid-recovery option that offers a sense of active intervention. In contrast, older adults, men, and rural residents were significantly less likely to opt for endoscopic surgery, possibly due to lower concern for cosmetic outcomes, stronger trust in traditional open procedures, and limited acceptance of newer techniques. Patients from rural areas, with lower income, education, and weaker social support, were more likely to reject AS, reflecting limited health management capacity, reduced access to information, and greater discomfort with uncertainty—often preferring immediate treatment for reassurance (38). This may indicate a lack of confidence in the adequacy of AS within local healthcare settings. Notably, unlike findings from Nickel et al. (23) in Australia, our study observed that Chinese patients with higher HL and SE were more inclined towards early invasive treatment rather than AS. This may reflect a cultural context in which cancer is viewed as an urgent threat, and non-interventionist approaches like monitoring are often misunderstood as neglect, making it difficult for patients to tolerate prolonged uncertainty and psychological burden.

### 4.3 Recommendations

To optimise treatment decision-making for low-risk PTMC patients in China, it is crucial to balance patient concerns about long-term outcomes with the practicality of treatment options. Given the strong preference for long-term reassurance and the willingness to accept more invasive treatments, health care providers should focus on clear communication regarding the benefits and risks of each treatment, emphasising both long-term and short-term considerations. For patients preferring minimally invasive treatments like TA, more widespread education and access to these options should be promoted. On the other hand, to address mistrust of AS, efforts should be made to improve patient understanding of the safety and effectiveness of active surveillance, particularly in rural areas, by strengthening health care support systems, offering better counselling, and increasing access to information (39).

### 4.4 Limitations

This study has several limitations. First, the DCE methodology relies on hypothetical scenarios, which may not fully capture real-world decision-making. Second, excluding 16.4% of participants due to rapid survey completion, comprehension failures, or incomplete information may have introduced selection bias. Third, factors like cultural beliefs, prior healthcare experiences, and individual health status, which may also influence treatment preferences, were not included. Future research should involve a more diverse population and explore these additional factors to validate and extend the results.

## Data Availability

The raw data supporting the conclusions of this article will be made available by the authors, without undue reservation.
