# Supplementary material for: Treatment preferences in low-risk papillary thyroid microcarcinoma: a discrete choice experiment
Source: Front Public Health. 2025 Dec 15;13:1686729. doi: 10.3389/fpubh.2025.1686729 (PMC12745395; doi:10.3389/fpubh.2025.1686729)
Supplement: Supplementary file 1 [file Data_Sheet_1.docx]

# Supplementary Material

**Supplementary Table S1.** Attributes and Associated Levels used in the Discrete Choice Experiment

**Supplementary Table S2.** Example of Scenario Shown to Participants

**Supplementary Table S3.** Standardised Scale Measures Used in the Study

This supplementary material has been provided by the authors to give readers additional information about their work.

| **Attributes** | **Levels** | | |
| --- | --- | --- | --- |
|  | **Treatment Plan A**^1-6^ | **Treatment Plan B**7-1^4^ | **Active Surveillance**^15-18^ |
| Anesthesia+Scar | General anesthesia+neck scar*^a^* General anesthesia+axillary/chest scar*^b^*  General anesthesia+no visible scar*^b^* | Local anesthesia+no visible scar*^c^* | No anesthesia+no visible scar |
| Length of hospital stays*^d^* (day) | 2, 4, 6 | 0, 2, 4 | 0 |
| Risk of short-term complications*^e^* (%) | 10, 20, 30 | 5, 10, 15 | 0 |
| Risk of permanent voice change*^f^* (%) | 1, 5 | 0.1 | 0 |
| Risk of requiring lifelong thyroid replacement medication*^g^* (%) | 20, 40 | 0.1 | 0 |
| Risk of disease recurrence/progression within 10 years*^h^* (%) | 1, 3, 5 | 1, 5, 10 | 10, 15, 20 |
| Out-of-pocket costs for treatment*^i^* (CNY) | 5000, 10000, 15000, 20000 | 5000, 10000, 15000, 20000 | 0 |
| *a*Open partial thyroidectomy: Under general anesthesia, the surgical instruments are used to reach the thyroid lesion through a 5-10cm incision in the anterior neck, with scar in the neck after surgery. | | | |
| *b*Endoscopic partial thyroidectomy: Under general anesthesia, the surgical endoscopes are used to reach and remove thyroid lesions through several 0.5-1 cm incisions, with access routes including the pre-sternal, axillary, subclavian, postauricular, or transoral approaches, without scar in the neck after surgery. | | | |
| *c*Thyroid thermal ablation: Under local anesthesia, the ablation needle reaches the thyroid gland through a 0.1-0.3cm pinhole in the neck and destroys the thyroid nodule with heat, without scar after ablation. | | | |
| *d*Length of hospital stays: The total number of days a patient is admitted to the hospital for surgical or ablative treatment, encompassing the preoperative and postoperative periods. | | | |
| eRisk of short-term complications: After partial thyroidectomy or thyroid thermal ablation, complications may occur, including dysphagia, drinking cough, bleeding, infection, subcutaneous ecchymosis, voice changes (hoarseness, low blunt), thyroid dysfunction, parathyroid dysfunction, etc. Most of these complications are short-term complications, lasting no more than 6 months. | | | |
| *f*Risk of permanent voice change: The likelihood of experiencing irreversible alterations in voice quality, pitch, or volume due to potential damage to the recurrent laryngeal nerve or other related structures during surgical or ablative treatment. | | | |
| *g*Risk of requiring lifelong thyroid replacement medication: The probability of needing continuous thyroid hormone replacement therapy after surgical or ablative treatment, usually resulting from significant reduction or complete loss of thyroid function. | | | |
| *h*Risk of Disease recurrence and progression within 10 years: This refers to the probability of disease worsening or recurring over a 10-year period, which includes the development of new nodules, recurrence of the original lesion following partial thyroidectomy or thyroid thermal ablation, tumor enlargement (an increase in diameter ≥3 mm or volume ≥50%) during active surveillance, the occurrence of lymph node metastasis and distant metastasis. In the event of the above situations, patients should consider further treatment options to control disease progression. | | | |
| *i*Out-of-pocket costs for treatment: It refers to the out-of-pocket cost for partial thyroidectomy or thyroid thermal ablation after reimbursement under basic medical insurance, excluding other expenses related to disease treatment, such as diagnostic tests and transportation fees. All patients included in the study were covered by basic medical insurance, with China's basic medical insurance coverage consistently exceeding 95%. | | | |

Supplementary Table S1. Attributes and Associated Levels used in the Discrete Choice Experiment

Supplementary Table S2. Example of Scenario Shown to Participants

Please read the following information and complete 10 choice tasks.

Assume that after a thorough examination, your thyroid nodule shows no lymph node metastasis in the neck or distant metastasis, and it is not classified as a poor prognosis subtype. Most patients with such nodules have a good prognosis. These nodules typically grow slowly and may even remain unchanged, allowing patients to live with them for a lifetime. You can now choose from the following three treatment options:

**Treatment Plan A**: Surgical removal of the thyroid nodule through traditional open surgery or endoscopic surgery. The local recurrence rate after surgery is 2% to 6%, and the disease-specific mortality rate is less than 1%.

**Treatment Plan B**: Insertion of a microwave needle or radiofrequency needle into the nodule to heat and destroy it. This includes treatments like microwave ablation and radiofrequency ablation, which can achieve results like surgery.

**Active Surveillance**: No immediate intervention for the thyroid nodule, but regular monitoring of its growth and changes until disease progression is observed, at which point removal or ablation may be performed. Disease progression includes an increase in the largest diameter of the thyroid nodule by ≥3 mm or an increase in nodule volume by ≥50% compared to the initial diagnosis, or the appearance of regional lymph node metastasis or distant metastasis.

**Note**: All treatments will incur medical costs and carry the risk of both short-term and long-term complications. After treatments, complications may occur, including difficulty swallowing, choking on liquids, bleeding, infection, subcutaneous bruising, voice changes (hoarseness, deepening), thyroid dysfunction, and parathyroid dysfunction. Most complications are short-term, but long-term complications (lasting more than 6 months) may also occur, such as permanent voice changes or the need for lifelong thyroid replacement medication. Patients who opt for active surveillance will also require treatment after disease progression.

Please complete the following choice tasks based on your personal preferences.

Which program do you prefer? [Single choice]

○Treatment Plan A ○Treatment Plan B ○Active Surveillance

| Attributes | Treatment Plan A | Treatment Plan B | Active Surveillance |
| --- | --- | --- | --- |
| Risk of disease recurrence/progression within 10 years | 1% | 10% | 10% |
| Out-of-pocket costs for treatment | 20000 CNY | 5000 CNY | None |
| Anesthesia+Scar | General anesthesia+neck scar | Local anesthesia+no visible scar | No anesthesia+no visible scar |
| Length of hospital stays | 2 Days | 2 Days | None |
| Risk of short-term complications | 30% | 10% | None |
| Risk of permanent voice change | 5% | 0.1% | None |
| Risk of requiring lifelong thyroid replacement medication | 20% | 0.1% | None |

Supplementary Table S3. Standardised Scale Measures Used in the Study

| Scales | Items | Form of variable values |
| --- | --- | --- |
| Health Literacy Scale-Short Form | How do you feel about finding treatment information for the illness you have? | 1=Very difficult, 2=Difficult, 3=Easy, 4=Very easy; from 4 to 16; Cronbach's α=0.9257 for the main study survey. |
|  | How do you feel about assessing the pros and cons of different treatment options? |  |
|  | How do you feel about finding information on how to cope with mental health issues? |  |
|  | How do you feel about determining the types of vaccines you might need to get vaccinated with? |  |
| General Self-Efficacy Scale | When faced with difficult tasks, I am confident that I can accomplish them. | 1=Strongly disagree, 2=Disagree, 3=Neutral, 4=Agree, 5=Strongly agree; from 3 to 15; Cronbach's α=0.9270 for the main study survey. |
|  | I will be able to successfully overcome many challenges. |  |
|  | I have confidence in my ability to effectively complete a wide range of tasks. |  |
| Perceived Social Support Scale | I can receive emotional help and support from my family when needed. | 1=Strongly disagree, 2=Disagree, 3=Slightly disagree, 4=Neutral, 5=Slightly agree, 6=Agree, 7=Strongly agree; from 3 to 21; Cronbach's α=0.7824 for the main study survey. |
|  | My friends can truly help me. |  |
|  | There are certain people (leaders, relatives, colleagues) in my life who care about my emotions. |  |
| Fear of Progression Questionnaire-Short Form | The potential progression of my illness causes me anxiety. | 1=Never, 2=Rarely, 3=Sometimes, 4=Often, 5=Always; from 12 to 60; Cronbach's α=0.9121 for the main study survey. |
|  | I get nervous before my doctor's appointments or regular check-ups. |  |
|  | I fear the pain associated with this illness. |  |
|  | The idea of reduced productivity at work due to my illness distresses me. |  |
|  | When anxious, I feel physical symptoms like a racing heart, stomachaches, and tension. |  |
|  | I worry that my illness may be transmitted to my children. |  |
|  | The prospect of having to rely on strangers for daily tasks fills me with anxiety. |  |
|  | I'm concerned that my illness may prevent me from enjoying my hobbies and interests. |  |
|  | I'm apprehensive about undergoing major treatments during the course of my illness. |  |
|  | I'm concerned about the potential harm medications may cause to my body. |  |
|  | I worry about the well-being of my family if something were to happen to me. |  |
|  | The thought of not being able to work due to my illness troubles me. |  |

# References

1. 任超,翟新法,渠海章,等.经胸乳入路腔镜手术治疗女性单侧甲状腺微小乳头状癌的临床价值分析.*中国医学文摘(耳鼻咽喉科学)*. 2023;38(04):50-52+71.

Ren C, Zhai XF, Qu HZ, et al. Clinical value analysis of trans-thoracic mammary approach laparoscopic surgery for female unilateral papillary thyroid microcarcinoma. *Chin Med Abstr (Otorhinolaryngol)*. 2023;38(04):50-52+71.

1. 许正强,吴兴桂,朱军华,等.经口腔前庭入路与经乳晕入路腔镜甲状腺切除术对甲状腺微小乳头状癌VAS评分、创伤-免疫指标及美观程度的影响.*现代生物医学进展*. 2023;23(11):2135-2138.

Xu ZQ, Wu XG, Zhu JH, et al. Comparison of the effects of trans-oral vestibular approach and trans-areolar approach laparoscopic thyroidectomy on VAS scores, trauma-immune indicators, and aesthetic outcomes in patients with papillary thyroid microcarcinoma. *Prog Modern Biomed*. 2023;23(11):2135-2138.

1. 张雪.经腋窝入路腔镜下甲状腺切除术的临床应用研究.*吉林大学*. 2022.

Zhang X. Clinical application of laparoscopic thyroidectomy through the axillary approach. *Jilin University*. 2022.

1. 王迎春.经胸乳入路腔镜甲状腺与开放手术在治疗分化型甲状腺癌的对比分析研究.*宁波大学*. 2021.

Wang YC. Comparison of trans-thoracic mammary approach laparoscopic thyroidectomy and open surgery in the treatment of differentiated thyroid carcinoma. *Ningbo University*. 2021.

1. 甄卫东.无充气经腋窝入路腔镜甲状腺手术的应用研究.*蚌埠医学院*. 2021.

Zhen WD. Application of non-inflated axillary approach laparoscopic thyroid surgery. *Bengbu Medical College*. 2021.

1. 李想.无充气腋窝入路腔镜下甲状腺癌根治术与传统开放性手术治疗单侧甲状腺乳头状癌近期疗效的临床研究.*安徽医科大学*. 2021.

Li X. Clinical study on the short-term efficacy of non-inflated axillary approach laparoscopic thyroid cancer radical surgery versus traditional open surgery for unilateral papillary thyroid carcinoma. *Anhui Medical University*. 2021.

1. 霍胜男.甲状腺乳头状癌微波消融治疗的临床研究.*郑州大学*. 2022.

Huo SN. Clinical study on microwave ablation treatment for papillary thyroid carcinoma. *Zhengzhou University*. 2022.

1. 王欣娅.超声引导下微波消融治疗甲状腺微小乳头状癌的疗效分析与评价.*青岛大学*. 2022.

Wang XY. Efficacy analysis and evaluation of ultrasound-guided microwave ablation for papillary thyroid microcarcinoma. *Qingdao University*. 2022.

1. 邝焕杰.超声引导下射频消融甲状腺乳头状癌后体积的变化.*郑州大学*. 2022.

Kuang HJ. Volume change of papillary thyroid carcinoma after ultrasound-guided radiofrequency ablation. *Zhengzhou University*. 2022.

1. 鞠定吾.射频消融和手术切除治疗甲状腺微小乳头状癌的临床疗效对比.*郑州大学*. 2022.

Ju DW. Comparison of clinical efficacy between radiofrequency ablation and surgical resection for papillary thyroid microcarcinoma. *Zhengzhou University*. 2022.

1. 张广旭.低危甲状腺微小乳头状癌的消融与手术的疗效对比.*吉林大学*. 2021.

Zhang GX. Comparison of efficacy between ablation and surgery in low-risk papillary thyroid microcarcinoma. *Jilin University*. 2021.

1. 逯泓智.甲状腺微小乳头状癌热消融治疗有效性及安全性分析.*吉林大学*. 2021.

Lu HZ. Analysis of the effectiveness and safety of thermal ablation for papillary thyroid microcarcinoma. *Jilin University*. 2021.

1. 翁俊.热消融对比传统手术治疗甲状腺微小乳头状癌效果的系统评价与Meta分析.*宁夏医科大学*. 2021.

Weng J. Systematic review and meta-analysis of the effect of thermal ablation versus traditional surgery for papillary thyroid microcarcinoma. *Ningxia Medical University*. 2021.

1. 杜轲锋.超声引导下射频消融治疗甲状腺微小乳头状癌疗效及安全性分析.*郑州大学*. 2022.

Du KF. Efficacy and safety of ultrasound-guided radiofrequency ablation for papillary thyroid microcarcinoma. *Zhengzhou University*. 2022.

1. Ito Y, Miyauchi A, Inoue H, et al. An observational trial for papillary thyroid microcarcinoma in Japanese patients. *World J Surg*. 2010;34(1): 28-35.
2. Ito Y, Miyauchi A, Kihara M, et al. Patient age is significantly related to the progression of papillary microcarcinoma of the thyroid under observation. *Thyroid*. 2014;24(1): 27-34.
3. 刘文.积极监测代替立即手术管理中国人群低危甲状腺乳头状癌的安全性和有效性研究.*昆明医科大学*. 2022.

Liu W. Study on the safety and efficacy of active surveillance as an alternative to immediate surgical management for low-risk papillary thyroid microcarcinoma in the Chinese population. *Kunming Medical University*. 2022.

1. Le K, Jin L, Zhong F, et al. Tumor growth manifested in two-fifths of low-risk papillary thyroid microcarcinoma patients during active surveillance: data from a tertiary center in China. *Front Endocrinol*. 2024;15: 1359621.
2. Zhou J, Yin L, Wei X, et al. 2020 Chinese guidelines for ultrasound malignancy risk stratification of thyroid nodules: the C-TIRADS. *Endocrine*. 2020;70(2):256-279.
3. National Bureau of Statistics of China. 2023 Annual Statistical Report. Accessed January 1, 2024. https://www.stats.gov.cn/tjsj/ndsj/2023/indexch.htmv.
